# Suboptimal SARS-CoV-2−specific CD8^+^ T cell response associated with the prominent HLA-A*02:01 phenotype

**DOI:** 10.1073/pnas.2015486117

**Published:** 2020-09-10

**Authors:** Jennifer R. Habel, Thi H. O. Nguyen, Carolien E. van de Sandt, Jennifer A. Juno, Priyanka Chaurasia, Kathleen Wragg, Marios Koutsakos, Luca Hensen, Xiaoxiao Jia, Brendon Chua, Wuji Zhang, Hyon-Xhi Tan, Katie L. Flanagan, Denise L. Doolan, Joseph Torresi, Weisan Chen, Linda M. Wakim, Allen C. Cheng, Peter C. Doherty, Jan Petersen, Jamie Rossjohn, Adam K. Wheatley, Stephen J. Kent, Louise C. Rowntree, Katherine Kedzierska

**Affiliations:** ^a^Department of Microbiology and Immunology, Peter Doherty Institute for Infection and Immunity, University of Melbourne, Melbourne, VIC 3000, Australia;; ^b^Department of Hematopoiesis, Sanquin Research and Landsteiner Laboratory, Amsterdam University Medical Center, University of Amsterdam, 1066 CX Amsterdam, Netherlands;; ^c^Infection and Immunity Program, Biomedicine Discovery Institute, Monash University, Clayton, VIC 3800, Australia;; ^d^Department of Biochemistry and Molecular Biology, Biomedicine Discovery Institute, Monash University, Clayton, VIC 3800, Australia;; ^e^Department of Infectious Diseases, Launceston General Hospital, Launceston, TAS 7250, Australia;; ^f^School of Health Sciences and School of Medicine, University of Tasmania, Launceston, TAS 7248, Australia;; ^g^Department of Immunology and Pathology, Monash University, Melbourne, VIC 3800, Australia;; ^h^School of Health and Biomedical Science, Royal Melbourne Institute of Technology University, Melbourne, VIC 3000, Australia;; ^i^Centre for Molecular Therapeutics, Australian Institute of Tropical Health & Medicine, James Cook University, Cairns, QLD 4814, Australia;; ^j^Department of Biochemistry and Genetics, La Trobe Institute of Molecular Science, La Trobe University, Bundoora 3084 VIC, Australia;; ^k^School of Public Health and Preventive Medicine, Monash University, Melbourne, VIC 3004, Australia;; ^l^Infection Prevention and Healthcare Epidemiology Unit, Alfred Health, Melbourne, VIC 3004, Australia;; ^m^Department of Immunology, St. Jude Children’s Research Hospital, Memphis, TN 38105, USA;; ^n^Australian Research Council Centre of Excellence for Advanced Molecular Imaging, Monash University, Clayton 3800, VIC, Australia;; ^o^Institute of Infection and Immunity, Cardiff University School of Medicine, Cardiff CF14 4XN, United Kingdom;; ^p^Ausralian Research Council Centre of Excellence in Convergent Bio-Nano Science and Technology, University of Melbourne, Melbourne, VIC 3010, Australia

**Keywords:** CD8+ T cells, COVID-19, HLA-A*02:01, SARS-CoV-2 epitopes

## Abstract

An improved understanding of human T cell-mediated immunity in COVID-19 is important for optimizing therapeutic and vaccine strategies. Experience with influenza shows that infection primes CD8^+^ T cell memory to peptides presented by common HLA types like HLA-A2, which enhances recovery and diminishes clinical severity upon reinfection. Stimulating peripheral blood mononuclear cells from COVID-19 convalescent patients with overlapping peptides from severe acute respiratory syndrome coronavirus 2 (SARS-CoV-2) led to the clonal expansion of SARS-CoV-2−specific CD8^+^ and CD4^+^ T cells in vitro, with CD4^+^ T cells being robust. We identified two HLA-A*02:01-restricted SARS-CoV-2-specfic CD8^+^ T cell epitopes, A2/S_269–277_ and A2/Orf1ab_3183–3191_. Using peptide−HLA tetramer enrichment, direct ex vivo assessment of A2/S_269_^+^CD8^+^ and A2/Orf1ab_3183_^+^CD8^+^ populations indicated that A2/S_269_^+^CD8^+^ T cells were detected at comparable frequencies (∼1.3 × 10^−5^) in acute and convalescent HLA-A*02:01^+^ patients. These frequencies were higher than those found in uninfected HLA-A*02:01^+^ donors (∼2.5 × 10^−6^), but low when compared to frequencies for influenza-specific (A2/M1_58_) and Epstein–Barr virus (EBV)-specific (A2/BMLF_1280_) (∼1.38 × 10^−4^) populations. Phenotyping A2/S_269_^+^CD8^+^ T cells from COVID-19 convalescents ex vivo showed that A2/S_269_^+^CD8^+^ T cells were predominantly negative for CD38, HLA-DR, PD-1, and CD71 activation markers, although the majority of total CD8^+^ T cells expressed granzymes and/or perforin. Furthermore, the bias toward naïve, stem cell memory and central memory A2/S_269_^+^CD8^+^ T cells rather than effector memory populations suggests that SARS-CoV-2 infection may be compromising CD8^+^ T cell activation. Priming with appropriate vaccines may thus be beneficial for optimizing CD8^+^ T cell immunity in COVID-19.

## Significance

As the recall of CD8^+^ T cell memory promotes rapid recovery in, for example, influenza, we investigated circulating SARS-CoV-2−specific CD8^+^ T cells from COVID-19 patients. For two HLA-A*02:01 SARS-CoV-2−specific CD8^+^ T cell epitopes, we found that, while ex vivo frequencies of responding T cells were approximately fivefold higher than for pre−COVID-19 samples, they were ∼10-fold lower than for influenza or EBV-specific memory CD8^+^ T cells. Additionally, SARS-CoV-2−specific CD8^+^ T cells recovered from convalescent COVID-19 patients had an atypically high prevalence of stem cell memory, central memory, and naïve phenotypes. Might this unexpectedly low prevalence of classical effector memory T cells be a negative consequence of the infectious process that could be avoided by prior priming with an appropriately constituted vaccine?

The current severe acute respiratory coronavirus 2 (SARS-CoV-2) pandemic has, as of September 2020, infected more than 25 million people, caused at least 850,000 deaths (1), and paralyzed economies globally. Although the majority of infections are mild to moderate and short in duration, ∼12 to 18% of COVID-19 patients develop severe disease requiring hospitalization, ∼5% are critical (2–4), and others who are less severely affected, and even asymptomatic, may still have some underlying pathology (5). These are still early days, and there is much that remains unknown about both the innate and adaptive immune responses in COVID-19. An urgent need is to develop a better understanding so that any immunopathology can be managed, and vaccine design and immunotherapies optimized.

So far as adaptive immunity is concerned, we do know that SARS-CoV-2−specific antibodies can be found in ∼95% of convalescent COVID-19 patients (6, 7) and that titers determined in virus neutralization assays correlate well with spike protein-binding immunoglobulin (Ig) levels measured by ELISA (8, 9). High serum-neutralizing antibody titers tend to be more prominent in severe COVID-19, which could be characteristic of prolonged antigen stimulation due to delayed virus clearance. Otherwise, the duration of SARS-CoV-2−specific IgG persistence in serum is far from clear, and we have much to learn about the CD4^+^ and CD8^+^ T cell responses.

Virus-specific CD8^+^ T cells are generally thought to be involved in the elimination of virus-infected cell “factories” in the acute response to respiratory viruses with, where there is established CD8^+^ T cell memory, that response being enhanced in both rapidity and magnitude to provide a measure of protection against the development of severe disease following secondary virus challenge. Survivors of the 2002–2003 SARS outbreak still maintain CD4^+^ and CD8^+^ T cell populations reactive to the SARS-CoV-1 nucleocapsid protein (10), and evidence of sustained T cell memory has also been found for Middle East respiratory syndrome (MERS) (11). Furthermore, it is possible that there may be some cross-reactive T cell memory for COVID-19 in people who have been infected with these viruses and, perhaps, more broadly, with the previously circulating common cold coronaviruses (12).

For SARS-CoV-2, there is growing evidence that virus-specific T cells are indeed being generated. Our early COVID-19 case study showed that both CD4^+^ T-follicular helper cells and activated CD38^+^HLA-DR^+^CD8^+^ T cells appeared in the patient’s blood at 3 d prior to recovery, suggesting that they played a part in the resolution of COVID-19 (13). Recent communications from others also reported the presence SARS-CoV-2−reactive CD4^+^ and CD8^+^ T cells in both acute and convalescent COVID-19 patients (14, 15). More disturbing, however, is an analysis suggesting that at least a proportion of the SARS-CoV-2−specific CD8^+^ T cells recovered from peripheral blood may be showing as an “exhausted” phenotype (16). Clearly, it is a matter of urgency to develop a better understanding of the integrity of the acute CD8^+^ T cell response in COVID-19 and how this impacts disease outcome.

Here, we utilized a combination of peptide prediction and in vitro peptide stimulation with overlapping peptides from the spike (S), nucleocapsid (N), and membrane (M) proteins to identify two SARS-CoV-2 epitopes restricted by HLA-A*02:01 (A2/S_269_ and A2/Orf1ab_3183_) in individuals with COVID-19. Using peptide-HLA-I tetramers, we performed direct ex vivo tetramer enrichment to define the frequency and activation profiles of the responding SARS-CoV-2−specific CD8^+^ T cells in acute and convalescent COVID-19 patients and in prepandemic peripheral blood monocular cells (PBMCs), tonsil, and lung tissues from uninfected donors.

Our data establish that HLA-A*02:01−restricted SARS-CoV-2−reactive CD8^+^ T cells can be detected directly ex vivo in both COVID-19 patients and in immunologically naïve individuals. However, while SARS-CoV-2−specific CD4^+^ T cell responses were broadly comparable to those found previously for other viruses, virus-activated CD8^+^ T cells that recognize SARS-CoV-2 peptides presented by the common (at least in Caucasians) HLA-A*02:01 MHC-I glycoprotein both were at low prevalence and express a less than optimal (for virus elimination) phenotype. These findings raise a number of questions. Is this apparent CD8^+^ T cell response defect limited to these particular epitopes? If so, are HLA-A*02:01 individuals at higher relative risk? Alternatively, if this is a general effect, is the SARS-CoV-2 virus in some way subverting CD8^+^ T cell responsiveness? Perhaps COVID-19 may be one disease where an appropriately designed vaccine may do better than nature when it comes to generating a protective CD8^+^ T cell recall response.

## Results

### COVID-19 Patient Cohort and Uninfected Controls.

This study of 18 COVID-19 cases included one person who remained asymptomatic, 10 who were symptomatic but were cared for at home, and 7 who were admitted to hospital, including 2 requiring supplemental oxygen (*SI Appendix*, Table S1). Control cells were tested from another 17 uninfected individuals who formed a control group (*SI Appendix*, Table S2). All COVID-19 patients (median age 54 y, 55.6% females) seroconverted for SARS-CoV-2 antibodies by receptor-binding domain ELISA (17), and 12 were HLA-A*02:01−expressing individuals. As controls, we analyzed preexisting A2/CD8^+^ T cell responses in prepandemic PBMC and tonsil samples from 12 HLA-A*02:01−expressing subjects across three age groups: children (median age 9.5 y), adults (median age 51 y), and the elderly (median age 66.5 y), with 44% being female (*SI Appendix*, Table S2). Additionally, we tested preexisting A2/CD8^+^ T cell populations in lung tissues from five HLA-A2 individuals (median age 42 y).

### CD4^+^ and CD8^+^ T Cell Responses to SARS-CoV-2 Overlapping Peptide Pools.

**Fig. 1.**
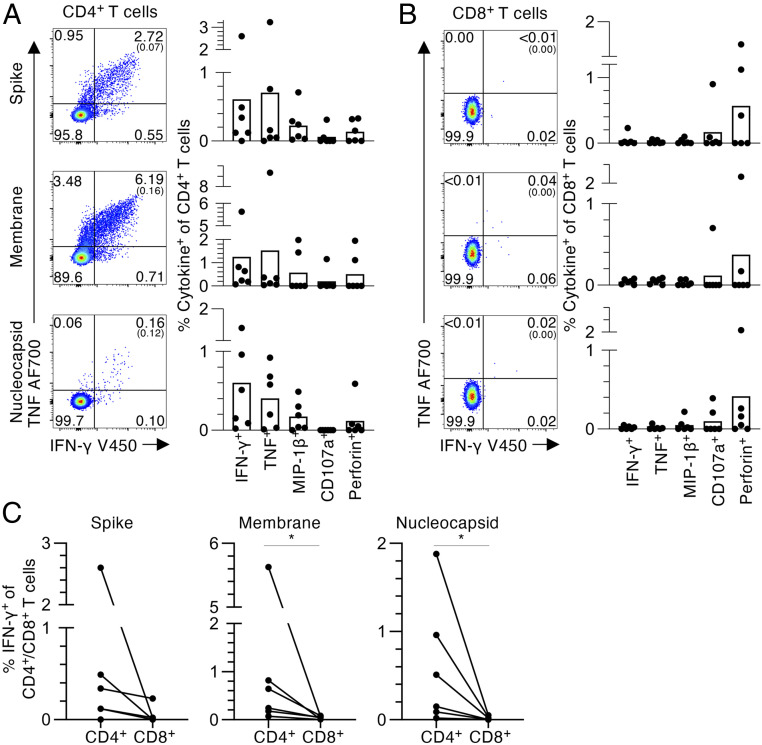
CD4^+^ and CD8^+^ T cell responses to SARS-CoV-2 overlapping peptide pools. (*A*) CD4^+^ and (*B*) CD8^+^ T cell responses to SARS-CoV2 S, M, and N peptide pools in convalescent COVID-19 individuals. (*i*) Representative fluorescence-activated cell sorter (FACS) plots showing IFN-γ and TNF staining of (*A*) CD4^+^ or (*B*) CD8^+^ T cell populations. (*ii*) Frequencies of IFN-γ^+^, TNF^+^, MIP-1β^+^, CD107a^+^ or perforin^+^ within the (*A*) CD4^+^ or (*B*) CD8^+^ T cells, with background staining subtracted (*n* = 6, mean). Background staining values are shown in brackets. Data points show individual COVID-19 convalescent subjects. (*C*) Paired frequencies of IFN-γ^+ ^CD4^+^ and CD8^+^ T cells for S, N, and M peptide pools. Statistical significance was determined with Wilcoxon matched-pairs signed rank test, *P < 0.05.

We first probed for SARS-CoV-2−specific CD4^+^ and CD8^+^ T cells in convalescent COVID-19 donors using a standard 6-h intracellular cytokine staining (ICS) assay using peptide pools containing 15mers, overlapping by 11 amino acids, which spanned the entire N and M proteins and selected regions of SARS-CoV-2 S protein. The PBMCs were stimulated with one peptide pool and expanded for 10 d before the assessment of SARS-CoV-2−reactive T cells by ICS for intracellular IFN-γ, TNF, and MIP-1β, plus staining for CD107a and perforin (Fig. 1 and *SI Appendix*, Fig. S1*A*) using individual peptide pools. The responding CD4^+^ T cells all stained for IFN-γ, TNF, MIP-1β, CD107a, and perforin, while the CD8^+^ T cells were predominately positive for perforin (Fig. 1 *A* and *B*). The CD4^+^ T cells showed significant staining for IFN-γ, with five out of six subjects generating IFN-γ^+^CD4^+^ T cells responses to at least one of the SARS-CoV-2 peptide N, M, or S pools, indicating that convalescent COVID-19 patients have solid SARS-CoV-2−specific CD4^+^ T cell immunity. However, while CD8^+^ T cells from 3/6 donors were perforin positive, evidence of modest IFN-γ^+^ activation for the CD8^+^ set was found in only one out of six donors. It thus seems that IFN-γ−producing SARS-CoV-2−specific CD4^+^ T cells expand to a much greater extent than the CD8^+^ set following in vitro peptide stimulation (Fig. 1*C*).

### Identification of SARS-CoV-2−Specific HLA-A*02:01−Restricted CD8^+^ T Cell Epitopes.

**Fig. 2.**
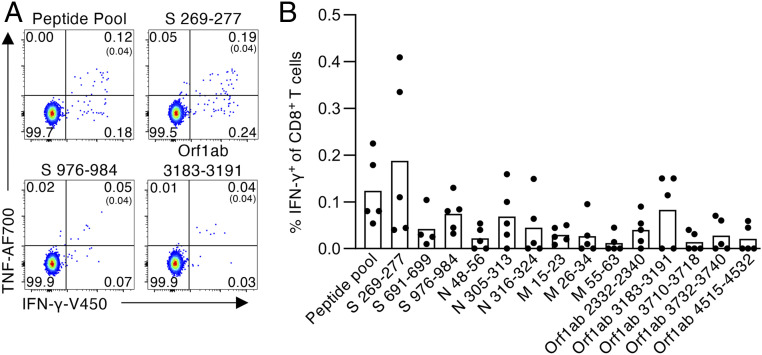
Identification of SARS-CoV-2−specific HLA-A*02:01−restricted CD8^+^ T cell epitopes. (*A*) Representative FACS plots of CD8^+^ IFN-γ/TNF staining after stimulation with the SARS-CoV-2 predicted peptide pool and individual S_269–277_, S_976–984_, and Orf1ab_3183–3191_ peptides. (*B*) Frequency of IFN-γ^+^ of CD8^+^ T cells for each SARS-CoV-2 peptide within the predicted peptide pool, with background staining subtracted (*n* = 5, mean). Peptide screen was performed in convalescent COVID-19 PBMCs after 10-d expansion in vitro.

Switching the focus to HLA-specific SARS-CoV-2 CD8^+^ T cell responses, we next identified CD8^+^ T cell specificities for HLA-A*02:01−expressing individuals. Using predicted HLA-A*02:01−binding SARS-CoV-2−derived peptides from the S, N, M, and Polyprotein1ab (Orf1ab) proteins (*SI Appendix*, Table S3; based on two prediction algorithms: NetCTLpan and NetMHCpan; accessed 27 March 2020), PBMCs from five HLA-A*02:01^+^ COVID-19 convalescent individuals were expanded in vitro with a pool of 14 predicted A2/SARS-CoV-2 peptides for 10 d, then restimulated with individual peptides in an ICS assay to determine peptide immunogenicity. Of the 14 peptides screened, S_269–277_ (YLQPRTFLL) generated the strongest CD8^+^IFN-γ^+ ^response (mean 0.19%, *n* = 5), with lesser responses being elicited for S_976–984_ (VLNDILSRL) and Orf1ab_3183–3191_ (FLLNKEMYL) (0.07% and 0.08%, respectively, mean, *n* = 5) (Fig. 2 and *SI Appendix*, Fig. S1*B*). Collectively, we identified one dominant and two subdominant A2/CD8^+^ T cell specificities for SARS-CoV-2.

Peptide sequence conservation analysis for these SARS-CoV-2 immunogenic peptides was extended to previously circulating coronaviruses. Reference protein sequences for SARS-CoV-1 and MERS plus the “common cold” human CoV (hCoV) strains 229E, HKU1, NL63, and OC43 were obtained from National Center for Biotechnology Information. Using the Virus Pathogen Resource (https://www.viprbrc.org/brc/home.spg?decorator=vipr), SARS-CoV2 S_269,_ S_976_, and Orf1ab_3183_ peptide sequences were compared to their respective protein sequences within each CoV strain (*SI Appendix*, Table S3). Our data showed that SARS-CoV-2/Orf1ab_3183_ and S_976_ lacked any sequence similarity to hCoV or MERS strains, but each shared 100% sequence identity with SARS-CoV-1/Orf1ab_3160–3168_ (FLLNKEMYL) and S_958–966_ (VLNDILSRL), respectively. SARS-CoV-2/S_269_ shared 78% and 67% sequence identity with MERS/S_317–325_ (KLQPLTFLL) and SARS-CoV-1/S_256–264_ (YLKPTTFML), respectively (positions that differ are underlined). Evidently, the A2/SARS-CoV-2 CD8^+^ T cell epitopes identified may be cross-reactive for SARS-CoV-1 and MERS, while they did not share homology with the common cold hCoVs that circulate in Australia.

### SARS-CoV-2−Specific A2/CD8^+^ T Cells Are at Low Frequency in COVID-19 Patients.

**Fig. 3.**
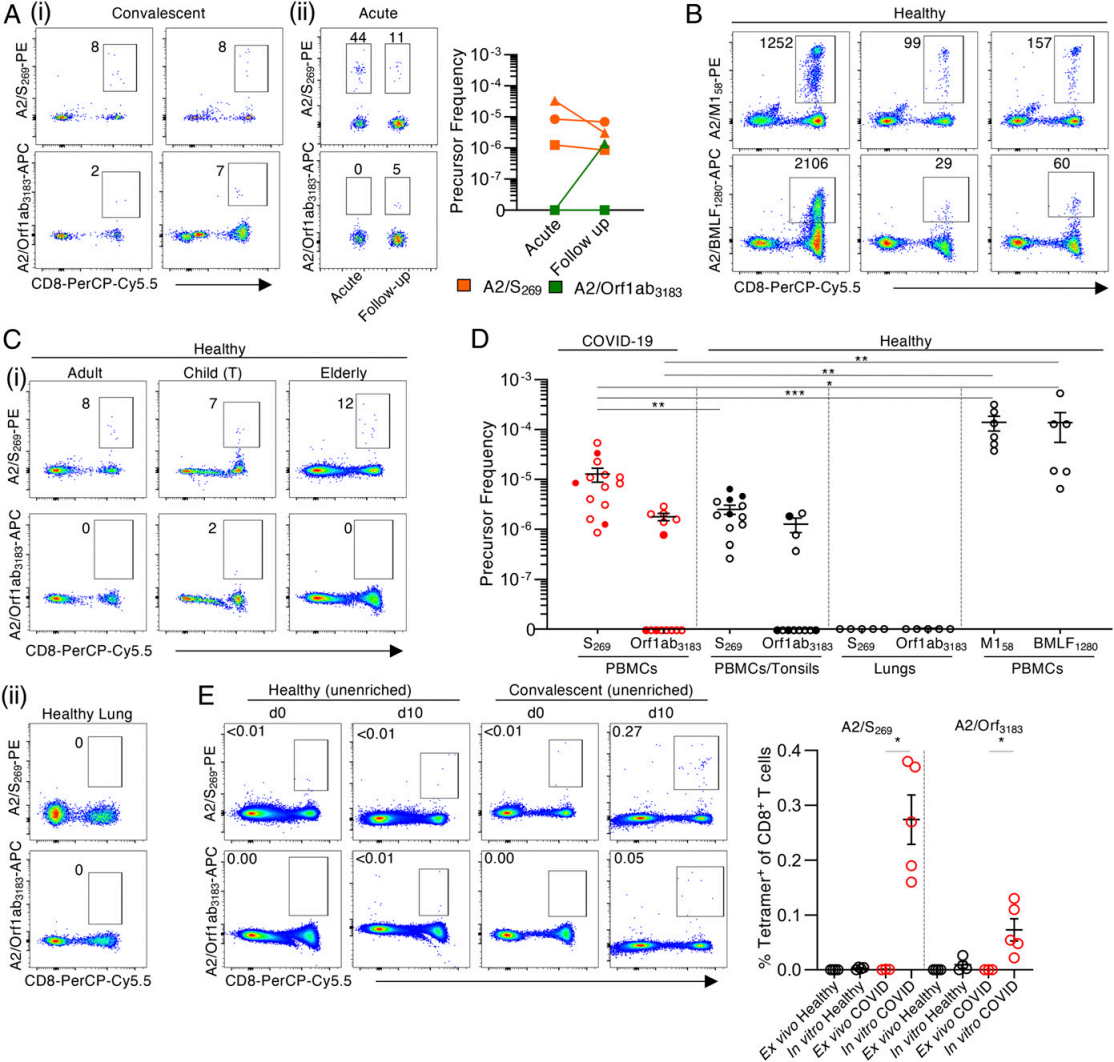
Low ex vivo frequency of SARS-CoV-2−specific A2/CD8^+^ T cell specificities in acute and convalescent COVID-19 patients. A2/S_269_^+^CD8^+^ and A2/Orf1ab_3183_^+^CD8^+^ T cells were identified directly ex vivo from healthy (pre−COVID-19) PBMCs, tonsils, and lungs, as well as acute and convalescent COVID-19 PBMCs by tetramer magnetic enrichment. (*A*) Representative FACS plots of A2/S_269_^+^CD8^+^ and A2/Orf1ab_3183_^+^CD8^+^ T cells from enriched samples of (*i*) convalescent and (*ii*) acute COVID-19 PBMCs. (*B*) Representative FACS plots of A2/M1_58_^+^CD8^+^ and A2/BMLF_1280_^+^CD8^+^ T cells from enriched healthy PBMCs. (*C*) Representative FACS plots of A2/S_269_^+^CD8^+^ and A2/Orf1ab_3183_^+^CD8^+^ T cells from (*i*) enriched adult and elderly PBMCs, and child tonsils (T) and (*ii*) tetramer staining of human lung tissue. (*D*) A2/CD8^+^ T cells precursor frequencies were calculated for A2/S_269_^+^CD8^+^, A2/Orf1ab_3183_^+^CD8^+^, A2/M1_58_^+^CD8^+^, and A2/BMLF_1280_^+^CD8^+^ T cells enriched from either PBMCs or tonsils, or stained in lungs. Dots represent individual donors. Means ± SEM are shown. Red dots are COVID-19 acute (closed circle) and convalescent (open circle) donors. Black dots are healthy adult or elderly PBMCs (open circle), or MNCs from child tonsils (closed circle). Donors with undetectable precursor frequencies of 0 are included on the graph to show the number of donors tested. These donors were not included in statistical analyses. Statistical significance was determined with two-tailed Mann−Whitney *U* test, *P < 0.05, **P < 0.01, ***P < 0.001. (*E*) Representative FACS plots and frequencies of A2/SARS-CoV-2^+^CD8^+^ T cells in the CD8^+^ population in healthy and convalescent donors on d0 and d10 of expansion. Dots represent individual donors. Statistical significance was determined using a two-tailed Mann−Whitney *U* test, *P < 0.05.

To further analyze the SARS-CoV-2−specific A2/CD8^+^ populations from COVID-19 patients, we generated tetramers for the A2/S_269_ and A2/Orf1ab_3183_ epitopes. Tetramer-associated magnetic enrichment (18, 19) was then used to determine the ex vivo frequencies of A2/S_269_^+^CD8^+^ and A2/Orf1ab_3183_^+^CD8^+^ T cells in acute and convalescent HLA-A*02:01^+^ cases. During the acute phase of COVID-19, A2/S_269_^+^CD8^+^ T cells were readily detected after ex vivo tetramer enrichment at a mean frequency of 1.44 × 10^−5^ (*n* = 3) in the CD8^+^ set, while the values for the A2/S_269_^+^CD8^+^ and A2/Orf1ab_3183_^+^CD8^+^ T cells from COVID-19 convalescents were 1.28 × 10^−5^ (*n* = 14) and 1.77 × 10^−6^ (*n* = 6), respectively (Fig. 3 *A* and *D*). There was no significant difference in the frequency of A2/S_269_^+^CD8^+^ T cells between acute and convalescent COVID-19 donors, while minimal A2/S_269_^+^CD8^+^ and A2/Orf1ab_3183_^+^CD8^+^ T cells were detected in either unenriched or flow-through samples (*SI Appendix*, Fig. S2). Indeed, while too few T cells were available to test other specificities concurrently for the COVID-19 patients, these frequencies of SARS-CoV-2−specific CD8^+^ T cells were significantly lower than those found for influenza A virus (IAV)-specific (1.39 × 10^−4^ for A2/M1_58_; *n* = 6) and Epstein–Barr virus (EBV)-specific (1.38 × 10^−4^ for A2/BMLF_1280_; *n* = 6) memory T cell populations from uninfected controls (Fig. 3 *B* and *D*), and as per previous publications (19, 20).

Are SARS-CoV-2−specific CD8^+^ T cells present in uninfected people? Using ex vivo tetramer enrichment with prepandemic PBMC, tonsil, and lung samples taken from HLA-A*02:01−expressing uninfected individuals (Fig. 3 *C*, i and *D*), naïve SARS-CoV-2−specific CD8^+^ T cells directed at A2/S_269_ were detected in all of the PBMC and tonsil samples (*n* = 12), while CD8^+^ T cells directed at A2/Orf1ab_3183_ were found in only 33% of individuals (*n* = 12), and the lung tissues were uniformly negative (Fig. 3 *C*, ii and *D*). Both the A2/S_269_^+^CD8^+^ and A2/Orf1ab_3183_^+^CD8^+^ were found over a broad range of ages (A2/S_269_: 5 y to 68 y; A2/Orf1ab, 11 y to 65 y). Moreover, the A2/S_269_^+^CD8^+^ T cell frequency of 2.5 × 10^−6^ (mean, *n* = 12) in pre−COVID-19 healthy individuals was significantly lower than that found for COVID-19−exposed individuals (*P* = 0.0064; Fig. 3*D*). It thus seems that the A2/S_269_^+^CD8^+^ T cells are indeed being activated and clonally expanded during SARS-CoV-2 infection. In contrast, there was no significant difference in frequencies for the A2/Orf1ab_3183_^+^CD8^+^ T cells from the prepandemic and COVID-19 groups (*P* = 0.4121) (Fig. 3*D*).

To further probe the responsiveness of A2/SARS-CoV-2 CD8^+^ T cells from uninfected versus convalescent COVID-19 donors, PBMCs or tonsil cells were stimulated with the S_269_ and Orf1ab_3183_ peptides and cultured in vitro for 10 d. In prepandemic “naïve” subjects, no evidence of proliferation in culture was found for the A2/S_269_^+^CD8^+^ or A2/Orf1ab_3183_^+^CD8^+^ sets (Fig. 3*E*). In contrast, both the A2/S_269_^+^CD8^+^ and A2/Orf1ab_3183_^+^CD8^+^ T cells from the COVID-19 donors increased significantly in numbers (*P* = 0.0357; Fig. 3*E*). Evidently, the SARS-CoV-2/CD8^+^ T cells from COVID-19 individuals (but not those from SARS-CoV-2 naïve subjects) were primed by SARS-CoV-2 and are thus, at least under in vitro conditions, capable of clonal expansion.

### Activation Profiles of SARS-CoV-2−Specific A2/CD8^+^ T Cells Directly Ex Vivo.

**Fig. 4.**
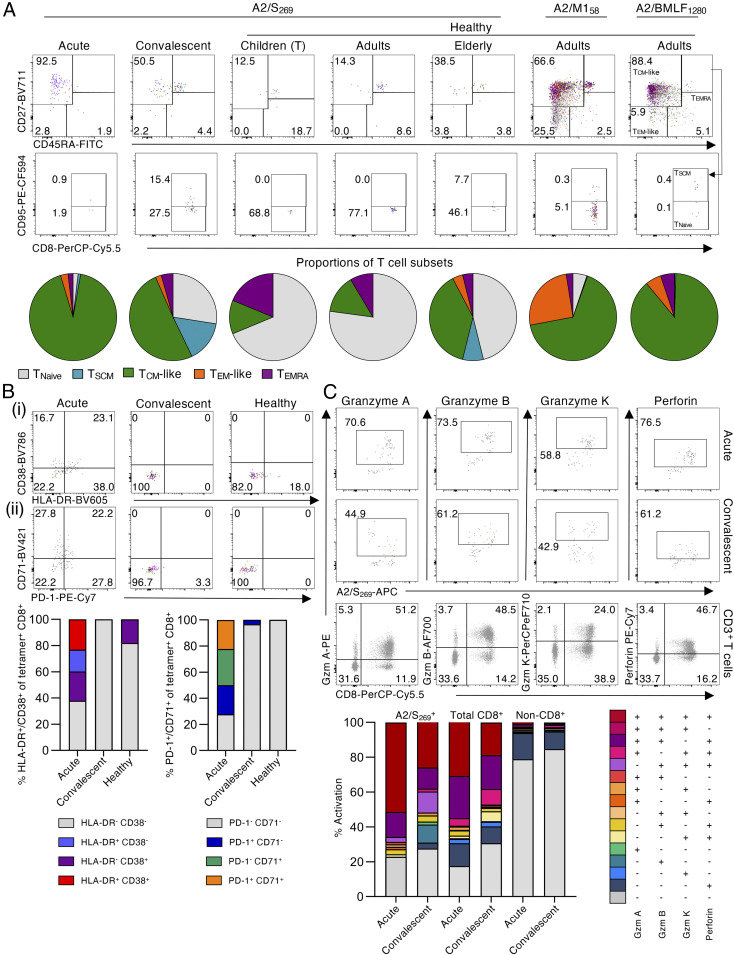
Ex vivo activation profiles of SARS-CoV-2−specific A2/CD8^+^ T cells in COVID-19 subjects. (*A*) Overlaid FACS plots of A2/S_269_^+^CD8^+^ T cells from acute COVID-19 (*n* = 3), convalescent COVID-19 (*n* = 11), healthy children (tonsils) (*n* = 4), healthy adults (*n* = 4), or healthy elderly donors (*n* = 4) show T_Naïve_ (CD27^+^CD45RA^+^CD95^−^), T_SCM_ (CD27^+^CD45RA^+^CD95^+^), T_CM_-like (CD27^+^CD45RA^−^), T_EM_-like (CD27^−^CD45RA^−^), and T_EMRA_ (CD27^−^CD45RA^+^) subsets. Pie charts display the proportion of each phenotype subset based on the combined data per each COVID-19 or healthy donor group. Overlaid FACS plots of A2/M1_58_^+^CD8^+^ and A2/BMLF_1280_^+^CD8^+^ T cell memory phenotypes from healthy adults are also shown. (*B*) Overlaid FACS plots and combined frequencies of A2/S_269_^+^CD8^+^ T cells showing (*i*) HLA-DR and CD38 or (*ii*) PD-1 and CD71 activation markers for acute (*n* = 3), convalescent (*n* = 11) and healthy donors (*n* = 12). (*C*) Overlaid FACS plots and combined frequencies of A2/S_269_^+^CD8^+^ T cells showing granzyme A, B, and K, and perforin staining for acute (*n* = 2) and convalescent (*n* = 3) donors. Representative FACS plots from one donor showing granzymes A, B, and K, and perforin of the total CD3^+^ T cell population. Combination gating was used to determine the frequency of cells with one to four functions for A2/S_269_^+^CD8^+^, total CD8^+^, or non-CD8^+^ T cells. Graphed data across multiple COVID-19 acute, COVID-19 convalescent, or naïve subjects were combined for the activation and phenotypic analyses of A2/S_269_ CD8^+^ T cells.

The activation profiles of A2/S_269_^+^CD8^+^ T cells tested directly ex vivo from acute and convalescent patients were assessed by CD27, CD45RA, and CD95 staining to determine the prevalence of the naïve (T_Naïve_) (CD27^+^CD45RA^+^CD95^−^), stem cell memory (T_SCM_) (CD27^+^CD45RA^+^CD95^+^), central memory (T_CM_)-like (CD27^+^CD45RA^−^), effector memory (T_EM_)-like (CD27^−^CD45RA^−^), and effector memory CD45RA (T_EMRA_) (CD27^−^CD45RA^+^) subsets (Fig. 4*A*). Acute COVID-19 donors displayed the highest proportion (mean of 92%) of T_CM_-like A2/S_269_^+^CD8^+^ T cells and a low proportion of T_EM_-like CD8^+^ T cells. The A2/S_269_^+^ CD8^+^ T cells from the convalescent versus acute subjects had a lower prevalence of T_CM_-like (mean of 50%) cells, and larger proportions of the T_Naïve_ (mean of 27%) and T_SCM_ (mean of 15%) sets, indicating that A2/S_269_^+^CD8^+^ T cells expressing the optimally responsive T_CM_ phenotype fall off rapidly in blood sampled after the infection has resolved. Conversely, the majority of A2/S_269_^+^CD8^+^ T cells within prepandemic children and adults were naïve (T_Naïve_; mean of 68% and 77%, respectively), while this subset was less prominent (mean of 46%) in the elderly. Interestingly, older, uninfected people had a mean of 38% T_CM_-like A2/S_269_^+^CD8^+^ T cells, similar to the frequency found for COVID-19 convalescents (mean of 50%), but less than that for IAV A2/M1_58_ (mean of 66%).

The expression profiles for HLA-DR, CD38, PD-1, and CD71 were also determined for tetramer^+^ A2/S_269_^+^CD8^+^ T cells from the COVID-19 patients (Fig. 4*B*). Only T cells from acutely infected donors were positive for these activation markers, with the majority coming from one individual (COVID-19 #2). In contrast, the A2/S_269_^+^CD8^+^ T cells from prepandemic and COVID-19 convalescent subjects were characterized by minimal levels of HLA-DR^+^CD38 and PD-1^+^CD71^−^, suggesting that, while the A2/S_269_^+^CD8^+^ set can be activated during the acute phase of the infection, it does not persist into short-term memory. Overall, our data suggest that naïve A2/SARS-CoV-2−specific CD8^+^ T cells can indeed be expanded approximately fivefold and activated during the acute phase of COVID-19 but that, atypically for what we know for other readily resolved infections like influenza, both the extent of T cell proliferation and the persistence of activated T cells in the blood is low for (days 37 to 101 post disease onset) convalescent individuals.

To further investigate the suboptimal activation of SARS-CoV-2−specific CD8^+^ T cells in COVID-19, the killing capacity of A2/S_269_^+^CD8^+^ T cells was assessed by staining for granzyme A, B, and K, and perforin directly ex vivo. Surprisingly, the majority of A2/S_269_^+^CD8^+^ T cells at both acute (mean of 77.2%) and convalescent (mean of 72.4%) stages of COVID-19 expressed three to four cytotoxic granzymes/perforin (Fig. 4*C* and *SI Appendix*, Fig. S3), indicating their activation status. However, a similarly high expression level of granzymes/perforin was also found on the majority of total CD8^+^ T cells (69 to 82.5%), as per our previous case report (13), but not on non-CD8^+^ T cells (mean of 15 to 21%). As it is highly unlikely that ∼80% of all CD8^+^ T cells in the peripheral blood during primary SARS-CoV-2 infection were antigen specific (even if directed at several CD8^+^ T cell epitopes), this suggests that a high proportion of CD8^+^ T cells are activated via some “bystander” mechanism during acute/convalescent COVID-19. The consequences, if any, of this effect for TCR-mediated activation merit further investigation.

## Discussion

As the research community drives forward to design and evaluate novel vaccines and immunotherapies for COVID-19, concurrent efforts directed at understanding how immunity works in this disease process are largely focused on patient studies. Applying our established expertise in the analysis of T cell-mediated immunity, we found here that the CD4^+^ “helper” T cell response looks relatively normal when compared with what happens in, for example, people who have been infected with an IAV. However, when it comes to the virus-specific CD8^+^ T cells that play an important role in ameliorating disease severity and driving recovery in other respiratory infections, our findings for COVID-19 are less encouraging. Although we were able to identify two SARS-CoV-2−specific CD8^+^ T cell epitopes associated with the ubiquitous (in Caucasian) HLA-A*02:01 MHC-I glycoprotein (A2/S_269–277_ and A2/Orf1ab_3183–3191_) and found evidence for T cell responsiveness, the results were not what we expected.

Our findings show that, while “early memory” CD8^+^ T cells can be detected in convalescent HLA-A*02:01 COVID-19 patients at frequencies approximately fivefold higher than those from prepandemic samples, the SARS-CoV-2−specific response was ∼10-fold lower than that found regularly for CD8^+^ T cells directed at IAV or EBV epitopes. In general, there was an overrepresentation of SARS-CoV-2−specific tetramer^+^CD8^+^ T cells expressing cell surface phenotypes that are considered to be characteristic of “stem cell memory” and naïve precursor status, suggesting that the infectious process is, in some way, limiting both clonal expansion and differentiation of the “classical” effector and central memory sets. An alternative explanation is, of course, that T cell effectors are being generated but are localized to, and perhaps “consumed in” (driven to apoptosis?) sites of virus-induced pathology.

Even so, it is the case that SARS-CoV-2−specific CD8^+^ T cells were found in all COVID-19 acute and convalescent donors, and in stored prepandemic PBMC and tonsil samples (but not lung tissues) from HLA-A*02:01 children, mature adults, and the elderly. As the frequency of these naïve, prepandemic SARS-CoV-2−specific CD8^+^ T cells (∼2.5 × 10^−6^) was numerically comparable to that found for naïve HIV (Gag_77–85_, SLYNTVATL), cancer (Survivin_96–104_), or Hepatitis C Virus (NS3_1073_)-specific CD8^+^ T cell populations in healthy HLA-A*02:01^+^ individuals (19–21), both their presence and the fact that they were not readily expanded following in vitro stimulation suggests that they were not a product of prior exposure to some cross-reactive epitope. In fact, these are likely the naïve precursors that would be stimulated by appropriate prime-and-boost vaccine strategies.

Earlier experiments in a mouse model of SARS-CoV-1 showed that a conventional, CD8^+^ T cell-targeted prime-and-boost approach indeed established substantial pools of memory SARS-CoV-1−specific CD8^+^ T cells capable of driving protection against lethal SARS-CoV-1 infection (22). The fact that the frequencies of A2/S_269_^+^CD8^+^ T cells in COVID-19 patients increased approximately fivefold suggests that these SARS-CoV-2−specific CD8^+^ T cells proliferated, to some extent, during primary COVID-19, however not to the level of well-established memory CD8^+^ T cell populations directed at other viral epitopes like IAV-specific A2/M1_58_ and EBV-specific A2/BMLF_1280_. Further studies are obviously needed to understand why this is so. In addition, as our acquaintance with this novel CoV continues, we will be able to determine whether there is long-term survival (at least at >1 y) of SARS-CoV-2−specific CD8^+^ memory T cells following primary COVID-19 along with whether, in now healthy survivors, they can be activated and clonally expanded following challenge with an appropriate vaccine.

Surprisingly, the memory A2/S_269_^+^CD8^+^ T cell populations in convalescent subjects were dominated by stem cell memory, central memory, and naïve phenotypes, and lacked expression of the CD38, HLA-DR, PD-1, and CD71 activation markers. This is in stark contrast to the highly activated T_EM_ and T_EMRA_ profiles found ex vivo in both short-term (day 25) and long-term (7 mo) memory A2/M1_58_^+^CD8^+^ T cells following avian A/H7N9 influenza infection (23, 24). These minimal activation profiles for epitope-specific CD8^+^ T cells in early COVID-19 convalescence could possibly reflect suboptimal priming of A2/S_269_^+^CD8^+^ T cells in primary COVID-19. Furthermore, a recent study by Zhou et al. (25) demonstrated perturbed dendritic cell and T cell function in SARS-CoV2 infection. Impaired dendritic cell function might negatively impact antigen processing and presentation to CD8^+^ T cells, thus at least partially explaining the limited differentiation of SARS-CoV-2−specific CD8^+^ T cells observed here.

It remains unclear whether this is broadly representative of primary CD8^+^ T cell responses in COVID-19 or specific to the epitopes analyzed here. There is a possibility that there are other HLA-A*02:01−restricted immunodominant epitopes, or even immunodominant epitopes restricted by other HLAs in HLA-A*02:01^+^ COVID-19 patients. The A2/S_269_ epitope identified in our study was, however, also independently reported in a recent preprint (26), suggesting it is a common HLA-A*02:01 epitope. Moreover, it is also possible that CD8^+^ T cells directed toward other HLA-A*02:01−restricted epitopes might have expressed high levels of PD-1 and thus had an impaired capacity to expand in vitro due to their exhausted phenotype. Further identification of CD8^+^ T cell epitopes across a broad range of HLA class I alleles and SARS-CoV-2 proteins is needed to provide a more detailed landscape of CD8^+^ T cell responses in COVID-19, their ex vivo frequencies, and activation profiles. In-depth analysis of epitope-specific T cell responses in severe and critical cases is also essential if we are to understand whether the activation profiles of early CD8^+^ T cell memory reflect disease severity. And, as the range of candidate vaccines that are tested through phase 1 trials expands, it would also be of great benefit to determine whether the characteristics of memory CD8^+^ T cells generated in the absence of active infection look more optimal than those described here.

Stimulation with overlapping peptides led to the expansion of SARS-CoV-2−specific CD8^+^ and CD4^+^ T cells in vitro, although CD4^+^ T cells dominated the response. This might support, at least partially, the previous elegant study showing that CD4^+^ T cells but not CD8^+^ T cells were of a greater importance in primary SARS-CoV-1 infection, as depletion of CD4^+^ T cells (but not CD8^+^ T cells) led to delayed viral clearance from the lungs, associated with reduced neutralizing antibody and cytokine production (27). It is also important to note that the S peptide pool from Miltenyi Biotec used here spans only selected regions (304 to 338, 421 to 475, 492 to 519, 683 to 707, 741 to 770, 785 to 802, and 885 to 1273) rather than the entire protein; thus some CD8^+^ and CD4^+^ T cell responses could have been missed. Recent evidence revealed that Th2 and Th17 cytokine profiles in COVID-19 patients can be associated with differential disease outcomes (28). Our analyses focused on Th1 cytokine responses for CD4^+^ T cells, leaving Th2 and Th17 cytokine responses unknown. Different cytokine profiles of epitope-specific CD4^+^ T cells should be investigated in future studies, especially when SARS-CoV2−specific CD4^+^ T cell epitopes are identified.

Our early report on immunity to COVID-19 in some of Australia’s first patients suggested that broad and concomitant immune responses were associated with recovery from mild-to-moderate COVID-19 disease (13). The key immune populations detected included antibody-secreting cells, helper follicular T cells, and activated (CD38^+^HLA-DR^+^) CD8^+^ and CD4^+^ T cells, together with progressive increases in SARS-CoV-2−specific IgM and IgG antibodies. Subsequent studies confirmed the activation of both CD4^+^ and CD8^+^ T cells as indicated by cell-surface marker expression (14, 15). The present much more extensive yet focused analysis does, however, raise questions concerning the integrity of the epitope-specific CD8^+^ T cell response in COVID-19. Given the variation in disease outcome with this infection, that obviously merits much more detailed analysis.

## Methods

### Study Participants and Ethics Statement.

Thirty-five subjects were recruited into this study. Acute and convalescent COVID-19 subjects were recruited via the Alfred Hospital, University of Melbourne, or James Cook University. Seven of the donors were admitted to hospital during their active infection (*SI Appendix*, Table S1). Acute COVID-19 cases were admitted to the hospital ward, with two patients requiring oxygen support (*SI Appendix*, Table S1). Healthy donors were recruited via University of Melbourne or buffy packs obtained from the Australian Red Cross LifeBlood (*SI Appendix*, Table S2). Tonsils were obtained from healthy individuals undergoing tonsillectomy (Tasmania, Australia). Lung samples were obtained prior to the COVID-19 pandemic via the Alfred Hospital’s Lung Tissue Biobank. All blood and tonsil donors were HLA typed by Victorian Transplantation and Immunogenetics Service. Peripheral blood was collected in heparinized tubes, and PBMCs were isolated via Ficoll−Paque separation.

Experiments conformed to the Declaration of Helsinki Principles and the Australian National Health and Medical Research Council Code of Practice. Written informed consents were obtained from all blood donors prior to the study. Lung tissues were obtained from deceased organ donors after written informed consents from the next of kin. Written informed consents were obtained from participants’ parents or guardians for underage tonsil tissue donors. The study was approved by the Alfred Hospital (#280/14), The University of Melbourne (#2056689, #2056761, #1442952, #1955465, and #1443389), the Australian Red Cross Lifeblood (ID 2015#8), the Tasmanian Health and Medical (ID H0017479), and the James Cook University (H7886) Human Research Ethics Committees.

Cell lines and reagents, ICS, ex vivo tetramer enrichment, and phenotypic analysis are described in *SI Appendix*.

## Supplementary Material

Supplementary File

Supplementary File

## Data Availability

All study data are included in the article and *SI Appendix*.
