# Supplementary material for: Suboptimal SARS-CoV-2−specific CD8+ T cell response associated with the prominent HLA-A*02:01 phenotype
Source: Proc Natl Acad Sci U S A. 2020 Sep 10;117(39):24384–91. doi: 10.1073/pnas.2015486117 (PMC7533701; doi:10.1073/pnas.2015486117)
Supplement: Supplementary File [file pnas.2015486117.sapp.pdf]

PNAS
www.pnas.org

Suboptimal SARS-CoV-2-specific CD8+ T-cell response associated with the prominent HLA-A*02:01 phenotype

Jennifer R Habel[1], Thi H O Nguyen[1], Carolien E van de Sandt[1,2], Jennifer A Juno[1], Priyanka Chaurasia[3], Kathleen Wragg[1], Marios Koutsakos[1], Luca Hensen[1], Xiaoxiao Jia[1], Brendon Chua[1], Wuji Zhang[1], Hyon-Xhi Tan[1], Katie L Flanagan[4,5,6,7], Denise L Doolan[8], Joseph Torresi[1], Weisan Chen[9], Linda M Wakim[1], Allen C Cheng[10,11], Peter C Doherty[1,12*], Jan Petersen[3,13], Jamie Rossjohn[3,13,14], Adam K Wheatley[1,15], Stephen J Kent[1,15], Louise C Rowntree[1*] and Katherine Kedzierska[1*]

Corresponding author: Peter C Doherty
Email: pcd@unimelb.edu.au

This PDF file includes:

Supplementary text
Figures S1 to S3
Tables S1 to S3
Legends for Datasets S1

Other supplementary materials for this manuscript include the following:

Datasets S1

www.pnas.org/cgi/doi/10.1073/pnas.2015486117

Supplementary Information Text

METHODS

Cell lines and reagents

C1R.A*02:01 cells were maintained in RF-10 medium (RPMI-1640 with 10% heat-inactivated FCS (Gibco; Thermo Fisher Scientific)), with 0.3 mg/mL hygromycin-B (Life Technologies). Overlapping synthetic peptides spanning the entire Nucleocapsid (N) and Membrane (M) proteins, and selected regions of SARS-CoV2 Spike (S) were purchased from Miltenyi Biotec (PepTivator SARS-CoV-2 Prot_N (130-126-699), Prot_M (130-126-703) and Prot_S (130-126-701)) and reconstituted in 80% DMSO. Synthetic SARS-CoV2 peptides predicted to bind HLA-A*02:01 were purchased from GenScript and reconstituted in DMSO. Tetramers were generated from soluble, biotinylated HLA-A2 monomers. Briefly, HLA α-heavy chain with C-terminal BirA biotinylation motif and β2-microglobulin were expressed and purified as inclusion bodies in *E. coli*, solubilized in 6 M Guanidine HCl and refolded with either S_{269} or $Orf1ab_{3183}$ peptide, in buffer containing 50 mM Tris pH8, 3 M urea, 0.4 M Arginine, 2 mM oxidised Glutathione, 20 mM Glutathione, 2 mM EDTA, 10 mM PMSF and cOmplete™ protease inhibitor (Roche). Following dialysis in 10 mM Tris, HLA monomers were purified via DEAE and HiTrapQ ion exchange chromatography, and biotinylated with BirA ligase in 50 mM Bicine pH 8.3, 10 mM ATP, 10 mM magnesium acetate and 100 µm d-biotin. Following S200 gel permeation chromatography fully biotinylated HLA monomers were stored at -80 °C and conjugated to fluorescently-labeled streptavidins (SA), PE-SA or APC-SA (BD Biosciences) at an 8:1 monomer to SA molar ratio to form pMHC-I tetramers.

Intracellular cytokine staining (ICS)

PBMCs and tonsil samples were stimulated with either 0.6 nmol of overlapping SARS-CoV2 peptides or 1 µM A2/SARS-CoV2 predicted peptides for 10 days in RF-10 medium (+20 U/mL IL-2) (29). On d10, cells were stimulated with peptides for 6 hrs in the presence of GolgiPlug and GolgiStop (BD Bioscience) plus 10 U/mL IL-2, and the SARS-CoV2-reactive T cells quantified using anti-IFN-γ-V450, anti-TNF-AF700, anti-MIP-1β-APC, anti-CD107a-AF488 (BD) and anti-perforin-PE-Cy7 (BioLegend) ICS (30). $CD8^+$ T cells specific for A2/SARS-CoV2 epitopes were quantified using IFN-γ/TNF ICS with C1R.A*02:01 cells used as antigen-presenting cells.

Ex vivo tetramer enrichment and phenotypic analysis

Cells (1-86x10^6) were stained with A2/S_{269}-PE and A2/$Orf1ab_{3183}$-APC tetramers at 1:100 for 1 hr in MACS buffer (PBS with 0.5% BSA and 2 mM EDTA). PBMCs and tonsil samples were incubated with anti-PE and anti-APC microbeads (Miltenyi) and $tetramer^+$ cells were enriched using magnetic separation (18, 19). Cells were stained with anti-CD71-BV421, anti-CD4-BV650, anti-CD27-BV711, anti-CD38-BV786, anti-CD14-APC-H7, anti-CD19-APC-H7, anti-CD45RA-FITC, anti-CD8-PerCP-Cy5.5, anti-CD95-PE-CF594 and anti-PD1-PE-Cy7 (BD) and anti-CD3-BV510, anti-HLA-DR-BV605 (Biolegend) and Live/Dead near-infrared (Invitrogen) for 30 mins, washed, resuspended in MACS buffer and analysed by flow cytometry. Lung cells were stained with the following alterations; anti-CD69-BV421, anti-CD103-FITC (BioLegend), and anti-CD45RO-PE-Cy7 (Thermo Fisher Scientific). To detect granzymes and perforin, cells were stained with A2/S_{269}-APC and enriched, then surface stained with anti-CD27-BV711, anti-CD3-PE-CF594, anti-CD4-BV650, anti-CD45RA-FITC, anti-CD19-APC-H7, anti-CD14-APC-H7 (BD), anti-CD8-BV421 (Biolegend) and Live/Dead aqua (Invitrogen) and stained intracellularly with anti-Granzyme-B-AF700 (BD), anti-Perforin-PE-Cy7 (Biolegend), anti-Granzyme-A-PE and

anti-Granzyme-K-PerCP-efluor710 (eBiosceince) after fixation/permeabilization with eBioscience™ Foxp3/Transcription Factor Staining Buffer Set (Thermo Fisher Scientific). Samples were acquired on a BD LSRII Fortessa. Flow cytometry data were analyzed using FlowJo v10 software.

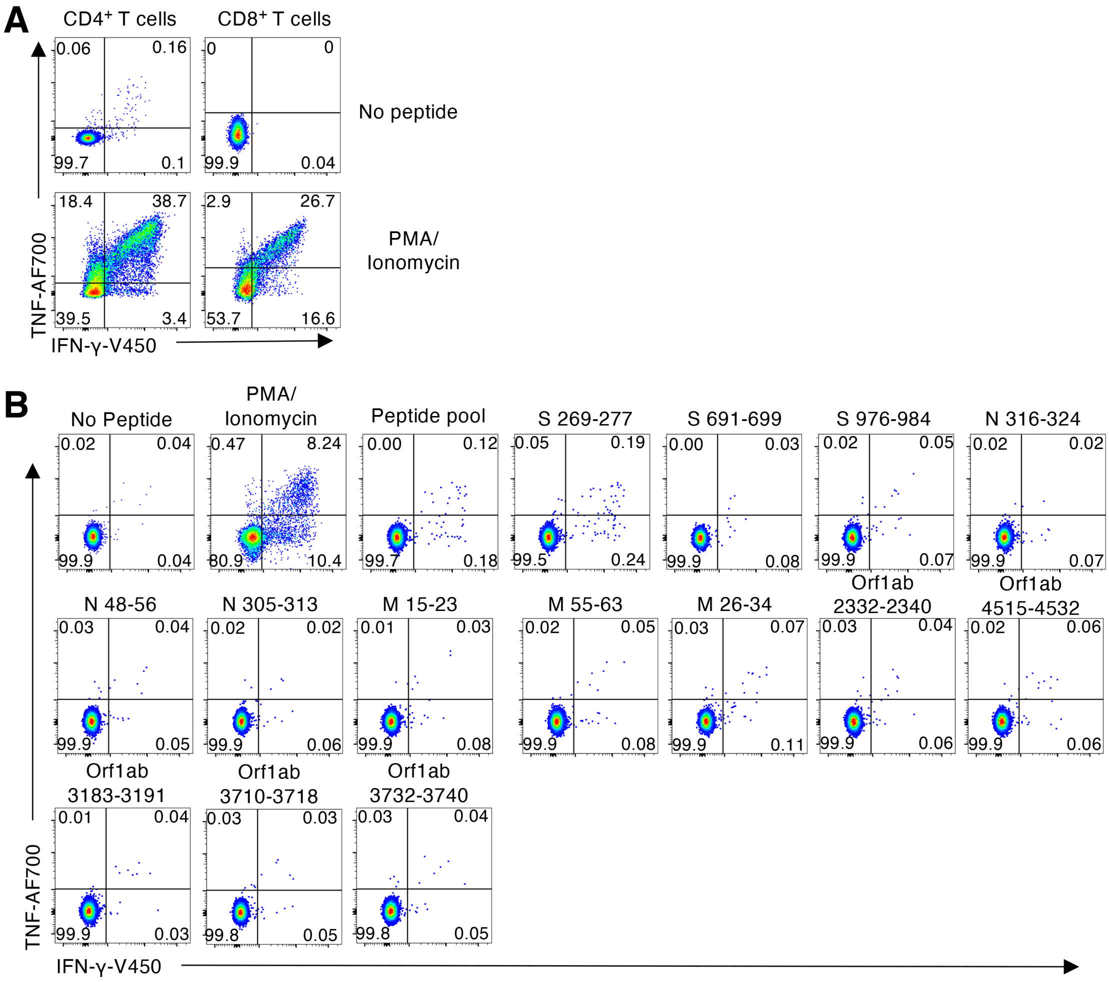

Fig. S1. (A) Representative FACS plots of IFN-γ/TNF staining of CD4⁺ and CD8⁺ T-cells stimulated with PMA-I (positive control) and DMSO (negative control) after d10 expansion *in vitro* with SARS-CoV-2 overlapping peptide pools. (B) Representative FACS plots after d10 culture with A2/SARS-CoV-2 predicted peptides. IFN-γ/TNF staining of CD8⁺ T-cells stimulated with PMA-I (positive control), DMSO (negative control), SARS-CoV-2 predicted peptide pool and individual A2/SARS-CoV2 peptides.

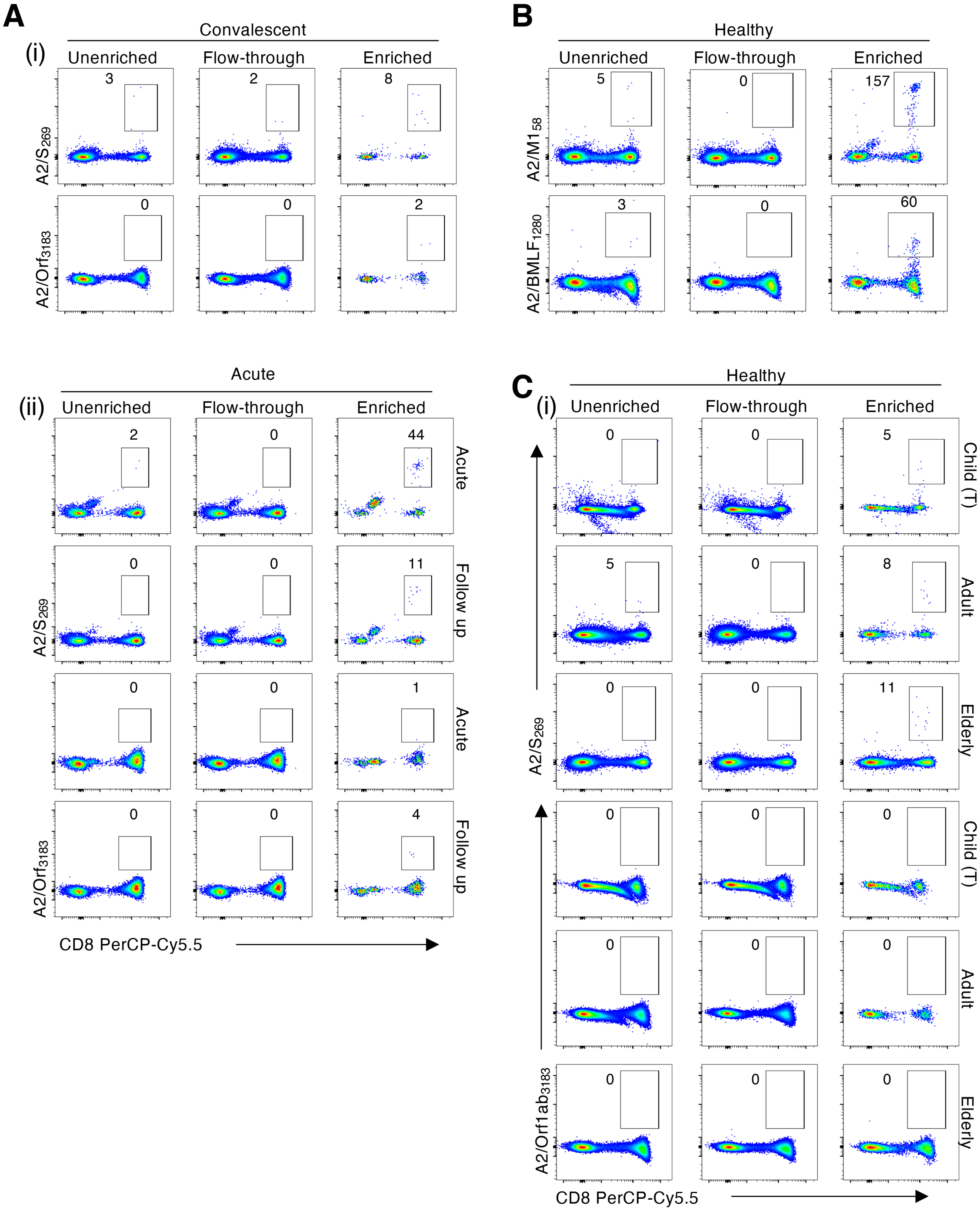

Fig. S2. (A) Representative FACS plots of A2/$S_{269}^{+}CD8^{+}$ and A2/Orf1ab$_{3183}^{+}CD8^{+}$ T-cells from unenriched, enriched and flow-through samples of (i) convalescent and (ii) acute COVID-19 PBMCs. (B) Representative FACS plots of A2/M$1_{58}^{+}CD8^{+}$ and A2/BMLF$_{1280}^{+}CD8^{+}$ T-cells from unenriched, enriched and flow-through samples of healthy PBMCs. (C) Representative FACS plots of A2/$S_{269}^{+}CD8^{+}$ and A2/Orf1ab$_{3183}^{+}CD8^{+}$ T-cells from unenriched, enriched and flow-through samples of adult and elderly PBMCs, and children tonsils (T).

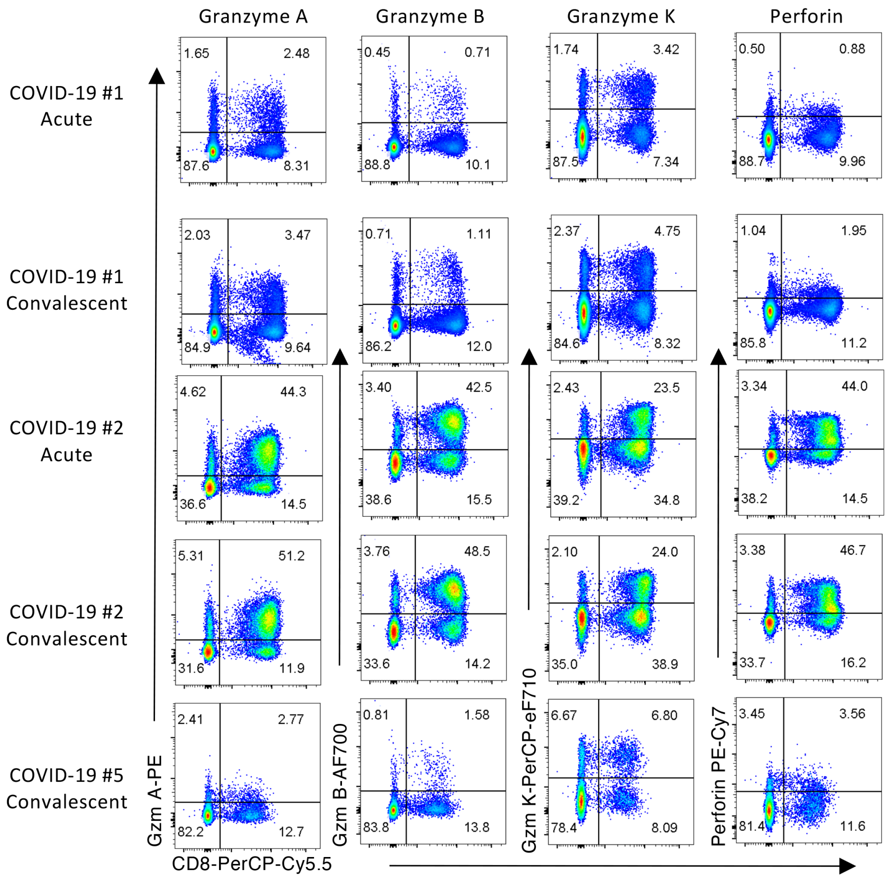

Fig. S3. FACS plots showing staining for granzymes A, B and K, and perforin of the $CD3^{+}$ population from acute and convalescent COVID-19 PBMCs.

Table S1. Demographic and clinical data for COVID-19 patients

Donor code	SARS-CoV-2 PCR	Seropositive SARS-CoV2*	Specimen	Age	Sex	Days from disease onset		Hospital (acute disease)	Oxygen (acute disease)	HLA-A	HLA-B	HLA-Cw
						Acute	Convalescent					
COVID-19 #1	positive	yes	blood	72	F	11	38	yes	yes	02:01	07:02, 15:18	07:02, 07:04
COVID-19 #2	positive	yes	blood	38	M	12	41	yes	no	02:01	15:01, 37:01	03:03, 06:02
COVID-19 #3	positive	yes	blood	73	F	10	44	yes	yes	01:01, 02:01	37:01, 40:01	03:03, 06:02
COVID-19 #4	positive	yes	blood	62	F	-	102	yes	no	03:01, 23:01	44:02; 44:03	04:01, 05:01
COVID-19 #5	positive	yes	blood	54	M	-	46, 101	yes	no	02:01, 31:01	38:01; 44:03	04:01, 12:03
COVID-19 #6	positive	yes	blood	56	F	-	101	yes	no	01:01	08:01, 57:01	06:02, 07:01
COVID-19 #7	positive	yes	blood	76	M	-	36	yes	no	01:01, 02:01	08:01, 35:01	04:01, 07:01
COVID-19 #8	positive	yes	blood	52	F	-	37	no	no	02:01, 31:01	40:01, 51:01	03:04, 05:01
COVID-19 #9	positive	yes	blood	22	F	-	42	no	no	02:01	44:02, 51:01	05:01
COVID-19 #10	positive	yes	blood	65	M	-	41	no	no	02:01, 24:02	07:02, 14:02	07:02, 08:02
COVID-19 #11	positive	yes	blood	56	M	-	55	no	no	02:01, 26:01	44:02	05:01, 06:02
COVID-19 #12	positive	yes	blood	31	M	-	48	no	no	01:01, 69:01	55:01, 57:01	01:02, 06:02
COVID-19 #13	positive	yes	blood	49	F	-	49	no	no	01:01, 11:01	08:01, 18:01	07:01
COVID-19 #14	positive	yes	blood	55	M	-	56	no	no	33:01, 68:01	14:02, 35:03	04:01, 08:02
COVID-19 #15	positive	yes	blood	24	F	-	45	no	no	02:01	39:01, 44:02	07:02, 07:04
COVID-19 #16	positive	yes	blood	51	F	-	93	no	no	02:01	07:02, 44:02	05:01, 07:02
COVID-19 #17	NT	yes	blood	54	F	-	94	no	no	02:01, 29:02	14:01, 44:03	08:02, 16:01
COVID-19 #18	positive	yes	blood	24	M	-	asymptomatic	no	no	01:01, 32:01	08:01, 14:01	07:01, 08:02

*seropositive by RBD ELISA; F: female; M: male; NT: not tested; Blood: heparinized blood

Table S2. Demographic data for healthy individuals

Donor code	Group	Specimen	Age	Sex	HLA-A	HLA-B	HLA-Cw
Healthy #1	Adult	Heparinized blood	30	F	02:01, 02:03	38:02, 40:01	07:02, 15:02
Healthy #2	Adult	Heparinized blood	52	M	02:01, 03:01	07:02, 55:02	03:03, 07:02
Healthy #3	Adult	Buffy pack	50	M	02:01, 29:02	44:02, 44:03	05:01, 16:01
Healthy #4	Adult	Buffy pack	58	F	01:01, 02:01	44:02	05:01
Healthy #5	Elderly	Buffy pack	72	NR	02:01, 11:01	07:02, 44:02	05:01, 07:02
Healthy #6	Elderly	Buffy pack	68	NR	01:01, 02:01	08:01, 14:01	07:01, 08:02
Healthy #7	Elderly	Buffy pack	65	NR	02:01, 30:01	13:02, 44:03	06:02, 16:01
Healthy #8	Elderly	Buffy pack	65	F	02:01, 03:01	07:02, 44:03	07:02, 16:01
Healthy #9	Child	Tonsil	5	M	02:01, 68:01	38:01, 44:02	05:01, 12:03
Healthy #10	Child	Tonsil	11	M	02:01, 31:01	07:02, 38:01	07:02, 12:03
Healthy #11	Child	Tonsil	16	F	01:01, 02:01	08:01, 44:03	07:01, 16:01
Healthy #12	Child	Tonsil	8	M	02:01	14:01, 57:01	06:02, 08:02
Healthy #13	Adult	Lung	61	M	02, 26	37, 55	06, 09
Healthy #14	Adult	Lung	65	M	02, 02	44, 57	05, 06, Cw5,6
Healthy #15	Adult	Lung	42	M	02, 26	15, 35	04, 05, Cw4,5
Healthy #16	Adult	Lung	41	F	02, 25	07, 08	07, 07
Healthy #17	Adult	Lung	30	M	02, 68	15, 51	03, 15

F: female; M: male; NR: not reported.

Table S3. A2/CD8+ SARS-CoV2 epitope prediction and conservation

Protein*	Sequence†	Residues	hCoV conservation	SARS-CoV1 conservation	MERS-CoV conservation
Spike	YLQPRTFLL	269-277	0%	67%	78%
	SIIAYTMSL	691-699			
	VLNDILSRL	976-984	0%	100%	0%
Nucleocapsid	GMSRIGMEV	316-324			
	NTASWFTAL	48-56			
	AQFAPSASA	305-313			
Membrane	KLLEQWNLV	15-23			
	WLLWPVTLA	55-63			
	FLFLTWICL	26-34			
Polyprotein1ab (Orf1ab)	ILFTRFFYV	2332-2340			
	TMADLVYAL	4515-4523			
	FLLNKEMYL	3183-3191	0%	100%	0%
	SMWALIISV	3732-3740			
	TLMNVLTLV	3710-3718			

*Protein sequences obtained from SARS-CoV-2 Reference Sequence (Refseq accession NC_045512.2; Protein ID YP_009724390.1, YP_009724397.2, YP_009724393.1, YP_ 009724389.1, respectively)

†Peptides provided by Genscript, Hong Kong, at >80% purity

Dataset S1 (separate file: Source data_Habel et al. xlsx). Source data for Figures 1-4.
